# Relationship Between Methane Emissions and Intestinal Methanogenic Microbiota in Micro‐Mini Pigs

**DOI:** 10.1111/asj.70130

**Published:** 2025-11-14

**Authors:** Maki Hirata, Eiko Nakashima, Iori Suenaga, Fumiki Morimatsu

**Affiliations:** ^1^ Bio‐Innovation Research Center Tokushima University Tokushima Japan; ^2^ Faculty of Bioscience and Bioindustry Tokushima University Tokushima Japan; ^3^ Center for Research Administration & Collaboration Tokushima University Tokushima Japan; ^4^ Sustainability Department NH Foods Ltd. Tokyo Japan

**Keywords:** gas monitoring chamber, methane, *Methanobrevibacter*, pig

## Abstract

Methane production in pigs has been less frequently studied in comparison to cattle. In this study, we developed an original greenhouse gas (GHG) monitoring system designed for pigs and explored the relationship between GHG emissions measured using this system and methane‐producing archaea in the porcine gut. The system comprises a semi‐closed monitoring chamber and a photoacoustic gas monitor capable of real‐time gas concentration monitoring. A gut microbiota analysis was conducted in parallel with the GHG measurements. Microbiota analysis revealed that the genus *Methanobrevibacter* dominated the intestinal microbiota of micro‐mini pigs, followed by the family Methanomethylophilaceae and genus *Methanosphaera*. Analysis of GHG emissions indicated that carbon dioxide emissions were correlated with body weight, while methane production was not associated with body weight, but rather with the abundance of the genus *Methanobrevibacter* in the gut. Methane production in the lower gastrointestinal tract of pigs was thought to be positively correlated with dietary fiber intake, and the composition of the intestinal microbiota may also play a role in methane generation. These findings will contribute to advancing research on GHG emission reductions in livestock production. However, a more comprehensive understanding of archaeal diversity requires further detailed analyses using methods targeted specifically at archaea.

## 1 Introduction

Greenhouse gas (GHG) emissions from anthropogenic activities, particularly industrial and agricultural production, are widely considered to be the primary drivers of global warming (Arora et al. 2018; Lamb et al. 2021). Livestock production contributes to global climate change via both direct and indirect factors (Cheng et al. 2022). Direct emissions include GHGs from enteric fermentation and manure management, while indirect emissions arise from feed production, and pasture establishment‐associated deforestation and land‐use changes (Gill et al. 2010). The primary GHGs emitted throughout the livestock production process include carbon dioxide, methane, and nitrous oxide (Haque 2018). However, carbon dioxide emissions from livestock are usually not regarded as a direct driver of climate change, as the animals consume plants that fix carbon dioxide through photosynthesis (Herrero et al. 2011). Therefore, methane and nitrous oxide represent the most influential GHGs emitted by animal production systems, given their substantial contribution to global warming. Nitrous oxide is primarily generated in manure and wastewater management. Several studies have been attempted to mitigate nitrous oxide emissions from pig production systems through wastewater treatment techniques to control nitrous oxide production, as well as adjusting feeding strategies that optimize nitrogen (N)—a source of nitrous oxide—to maintain the amino acid balance in their diets (Ogino et al. 2013; Yamashita et al. 2019). In contrast, methane is a key GHG that is associated with livestock production. Significant efforts have been made to mitigate methane emissions from livestock, which primarily include ruminants (Ku‐Vera et al. 2020). However, methane from non‐ruminant livestock, such as pigs and large herbivores, present in substantial numbers, should not be underestimated (Patra 2014). Although individual pigs emit less methane than ruminants, the global pig population exceeds one billion, and their cumulative methane emissions potently contribute to overall livestock‐related GHG emissions (Kim et al. 2024). Methane emissions from pigs have been less studied than those from ruminants, especially the composition and impact of gut microbial communities. A better understanding of the methane‐producing archaea in pigs may provide valuable insights to guide methane mitigation strategies for non‐ruminant livestock.

Methane is produced by methanogenic archaea in the digestive tract during food fermentation. Phylogenetic analyses of archaeal diversity in pigs have revealed that the genus *Methanobrevibacter* represents the most abundant methane‐producing archaea in colon digests, followed by *Methanosphaera*, and members of the order Methanomassiliicoccales (Mi et al. 2019). However, it remains unclear whether variations in the composition of methane‐producing archaea in the porcine intestinal tract influence the amount of methane generated. Moreover, the composition of methane‐producing archaea in the porcine digestive system varies with age and diet (Luo et al. 2017), and these variations may complicate efforts to accurately estimate methane production in pigs.

To address this knowledge gap, we developed a compact GHG monitoring system specifically designed for pigs, incorporating a photoacoustic gas monitor for real‐time measurement of carbon dioxide and methane concentrations. We verified the accuracy of this measurement chamber and analyzed the dynamics of carbon dioxide and methane emissions from pigs. We also assessed the correlation between methane emissions and intestinal methanogenic archaea. Micro‐mini pigs (MMPs) were used as experimental models due to their small body size and ease of handling under controlled conditions. Although not commonly used in commercial production, MMPs are practical and reliable models for exploratory research and system development.

## 2 Materials and Methods

### 2.1 Animals

All animal experiments were approved by the Institutional Animal Care and Use Committee of Tokushima University (Approval Number: T2022‐33). The animal care procedures and experiments were performed in accordance with the Guidelines for Animal Experiments of Tokushima University based on the Japanese Standards regulations relating to the Care and Keeping and Reducing Pain of Laboratory Animals. All experimental animals were born and housed in the Advanced Livestock System Development Center of Tokushima University. In total, 11 MMPs aged 9.5 ± 1.8 months (weight, 11.5 ± 1.7 kg) at the start of the study were used. The pigs were housed in a temperature‐controlled room (25 ± 2°C) under a 12‐h light/12‐h dark cycle with free access to water and were provided a specific diet formulated without antibiotics or probiotics (Japan Scientific Feeds Association, Japan).

### 2.2 Experimental Chamber

**FIGURE 1:**
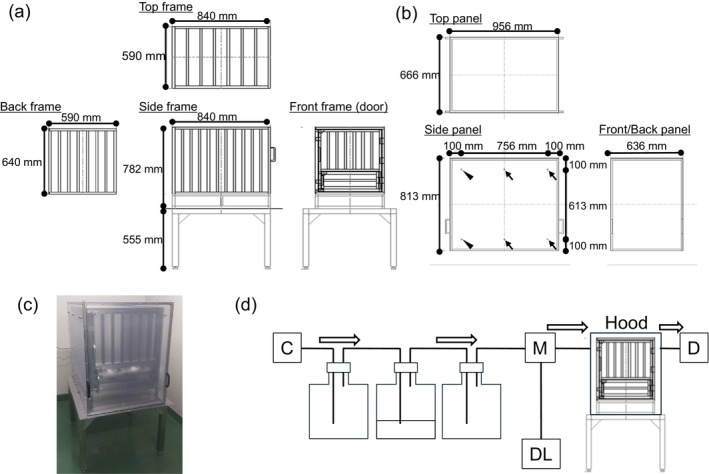
Illustrations and image of the cage and hood of the gas‐monitoring system. Schematic illustration of the (a) cage and (b) polycarbonate hood of the gas‐monitoring system. Arrowheads and arrows indicate the positions of the 10 and 4 mm connection ports, respectively. (c) Image of the gas‐monitoring system. (d) A schematic overview of the set‐up of the gas‐monitoring system. Arrows indicate air flow. C, compressor; M, mass flow meter; DL, data logger; D, detector (INNOVA photoacoustic gas monitor).

A custom‐built gas‐monitoring system was used in this study. The gas‐monitoring chamber involved a semi‐closed space created by covering a stainless‐steel cage with a polystyrene cover (Figure 1a–c). The cage was designed using square pipes to house one MMP during the test period (840 × 590 × 640 mm [L × W × H]). The bottom of the cage was raised and grated, allowing feces and urine to fall through. The polystyrene cover had 12 holes: four with a diameter of 10 mm and eight with a diameter of 4 mm. Bulkhead tube units were installed in all holes; a 10 mm partition was connected to an air inlet, and one of the 4 mm partitions was connected to a sampling port of Innova photoacoustic gas monitor 1512 (Advanced Energy, Denver, CO, USA). An air compressor was used to introduce air from the inlet at an approximate flow rate of 25 L/min. The flow rate was measured using a mass flow meter M‐50SLPM (Alicat Scientific, Tucson, AZ, USA) set between the air compressor and gas‐monitoring chamber (Figure 1d).

### 2.3 DNA Purification and 16S rRNA Gene Analysis

Fecal samples were collected directly from the rectums of MMPs. To minimize intra‐individual variation in fecal samples, two samples were collected before and after measurements, and the average of these sample values was used for subsequent analyses. The collected fecal samples were stored in a DNA stabilization buffer (4 M guanidine thiocyanate, 100 mM Tris–HCl [pH 9.0], and 40 mM EDTA) at 4°C until DNA extraction. The DNA extraction, purification, and next‐generation sequencing (NGS) analysis were conducted as described previously (Hirata et al. 2022). Briefly, fecal samples were diluted in TE buffer to a total volume of 600 μL, and then homogenized with glass beads (diameter, 0.15–0.21 mm) using a MagNA Lyser (Roche, Penzberg, Germany). Subsequently, 600 μL of phenol/chloroform/isoamyl alcohol and 100 μL of 10% sodium dodecyl sulfate were added, and the bead‐beating step was repeated. The final bead‐beating steps were performed after incubation at 70°C for 10 min. Subsequently, the samples were centrifuged at 20,380 ×*g* for 5 min at room temperature, and the upper layer was transferred to a 1.5‐mL tube. Following precipitation with isopropanol, the pellet was dissolved in 200 μL of TE buffer, after which a High Pure PCR Template Preparation Kit (Roche) was used for further DNA purification. The V3–V4 region of the bacterial 16S rRNA gene was amplified by PCR using the Illumina forward primer 5′‐AATGATACGGCGACCACCGAGATCTACAC (adaptor sequence) + barcode (eight bases) + ACACTCTTTCCCTACACGACGCTCTTCCGATCT (sequence primer) + CCTACGGGNGGCWGCAG‐3′ (341F), and reverse primer 5′‐CAAGCAGAAGACGGCATACGAGAT (adaptor sequence) + barcode (eight bases) + GTGACTGGAGTTCAGACGTGTGCTCTTCCGATCT (sequence primer) + GACTACHVGGGTATCTAATCC‐3′ (805R), as described previously (Hagihara et al. 2020). The resulting PCR products were then purified by gel extraction. Mixed samples were prepared by pooling approximately equal amounts of amplified DNA, and amplicon sequencing was performed by Azenta Life Sciences (Burlington, MA, USA) using the Illumina MiSeq platform with 2 × 300 bp paired‐end reads. QIIME2 (Version 2021.11, Quantitative Insights into Microbial Ecology, http://qiime2.org/) was used for sequence analysis (Bolyen et al. 2019), and the Silva 138.1 database was used to perform taxonomic classification. The relative abundance of each taxonomic group was calculated by dividing the read counts of identified sequences by the individual's total read number. The raw sequencing data generated in this study have been deposited in the DNA Data Bank of Japan (DDBJ) under the BioProject accession number PRJDB35533. These data will be made publicly available upon publication.

### 2.4 Data Collection

**FIGURE 2:**
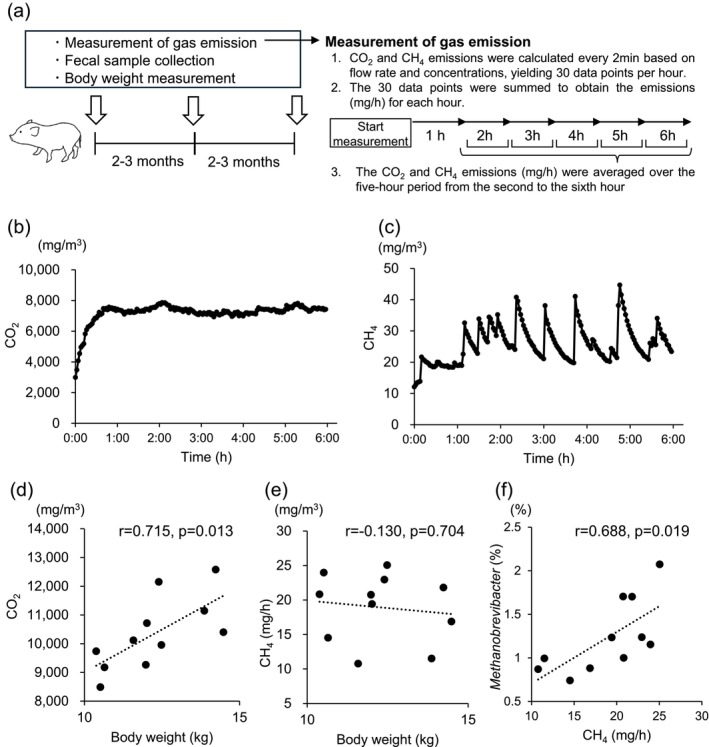
Greenhouse gas emission measurements and their relationship with body weight and gut microbiota. (a) The scheme of data collection and gas emission measurement process. Representative patterns of (b) CO_2_ and (c) CH_4_ concentration trends in the gas‐monitoring system. Correlation analysis between body weight and (d) CO_2_ or (e) CH_4_ emissions and between (f) CH_4_ emission and relative abundance of genus *Methanobrevibacter*. CO_2_ and CH_4_ emissions, body weight measurements, and gut microbiota analysis were performed three times for each of the 11 micro‐mini pigs, with analysis based on the average of these measurements. Relationships between two variables were statistically analyzed using Pearson correlation coefficients. r, correlation coefficient; CO_2_, carbon dioxide; CH_4_, methane.

Carbon dioxide and methane concentrations were measured using an Innova photoacoustic gas monitor (Maeda et al. 2013). Prior to the measurements, the inlet gas concentration was measured and used as the blank value. All experiments were initiated between 9:00 a.m. and 10:30 a.m., following the completion of feeding the animals. After introducing one MMP into the cage, the polycarbonate cover was placed and fastened using lashing belts. Data were collected at intervals of 2 min using Lumasence software for 6 h. Additionally, the flow rate data for inlet air were collected using a data logger GL240 (Graphtec Corporation, Yokohama, Japan) at 2‐min intervals. Carbon dioxide and methane emissions were calculated every 2 min based on the resulting concentrations and flow rates and subsequently summed up for each 1‐h interval (mg/h). The average carbon dioxide and methane emissions per hour during the 5‐h period from the second to the sixth hour were then calculated and used in subsequent analysis. Measurements using the gas‐monitoring system, fecal sample collections associated with these measurements, and the body weight measurements were conducted three times for each MMP at intervals of 2–3 months (Figure 2a).

### 2.5 Statistical Analysis

The carbon dioxide and methane emission values, gut microbiota analysis results, and the body weight data from the triplicate measurements were averaged for each individual and used in subsequent analysis. Pearson correlation coefficients were used to evaluate the relationship between body weight and carbon dioxide or methane emissions as well as between methane emissions and relative abundance of the genus *Methanobrevibacter*. Values of *p* < 0.05 were considered significant.

## 3 Results

The relative abundance of methane‐producing archaea detected in the feces of the MMPs used in this study is presented in Table 1. Five genera of methane‐producing archaea belonging to two families were identified; the genus *Methanobrevibacter* exhibited the highest relative abundance, followed by genus *RumEn_M2* and family Methanomethylophilaceae. Notably, the genera *Methanosphaera* and *Candidatus_Methanomethylophilus* were not detected in a few MMPs.

**TABLE 1:** Relative abundance of methane‐producing bacteria in micro‐mini pig gut microbiota.

Family	Genus	Prevalence in pigs (no.)	Relative abundance (%)^a^
Methanobacteriaceae	*Methanobrevibacter*	11/11	1.233 ± 0.421
	*Methanosphaera*	1/11	0.003
Methanomethylophilaceae	—	11/11	0.020 ± 0.010
	*RumEn_M2*	11/11	0.008 ± 0.005
	*Candidatus_Methanomethylophilus*	8/11	0.001 ± 0.001

^a^ Mean ± standard deviation. The relative abundance of each taxonomic group was calculated by dividing the read counts of identified sequences by the individual's total read number.

The representative patterns of carbon dioxide and methane concentration dynamics measured by the gas‐monitoring system are presented in Figure 2b,c. Carbon dioxide concentrations gradually increased following the initiation of measurement, stabilizing within 1 h and reaching a plateau after approximately 1 h. Consequently, data obtained 1 h after the initiation of measurement were used to evaluate the carbon dioxide and methane emissions. In contrast to carbon dioxide, the methane concentration fluctuated throughout the measurement period. The relationship between body weight and emissions of carbon dioxide and methane was subsequently examined. Body weight exhibited a significant positive correlation with carbon dioxide emission (Figure 2d), whereas no correlation was observed with the methane production (Figure 2e). However, a significant positive correlation was identified between *Methanobrevibacter* abundance in the gut microbiota and methane emission (Figure 2f).

## 4 Discussion

Respiration chambers represent a key tool for assessing animal energy metabolism and intestinal methane emissions in livestock. Several studies have utilized respiration chambers, along with head‐hoods and head‐boxes, to quantify methane production, particularly in cattle, which have attracted significant attention owing to their contribution to methane emissions (Roehe et al. 2016; Suzuki et al. 2021; Wallace et al. 2014). While several studies have used respiration chambers and other methods to examine the feed efficiency and energy requirements of pigs (Barea et al. 2010; Johnson et al. 2019; Le Goff et al. 2002), research on methane production in pigs remains limited. Christensen and Thorbek investigated methane production in fattening pigs using respiration measurements and reported that a 20–25 kg fattening pig generates approximately 1 L of methane per day (Christensen and Thorbek 1987). In our current study, an average methane production of approximately 20 mg/h equivalent to approximately 0.7 L over a 24‐h period, was obtained in MMPs with an average body weight of 11.5 kg. This value was slightly lower than that previously reported, which could be attributed to differences in body size, breed, or variations in fermentation capacity. Furthermore, for carbon dioxide, a review on measuring or estimating carbon dioxide exhalation in pigs proposed a formula for predicting the carbon dioxide emissions of fattening pigs (Philippe and Nicks 2015). Using this formula, carbon dioxide emission for a pig with an average body weight of 11.5 kg was estimated to be 0.55 kg per day. The average carbon dioxide emissions in this study were found to be 10,270 mg/h, which corresponds to approximately 0.25 kg of carbon dioxide per day. Considering that the MMPs generally have less muscle mass than that of fattening pigs with a similar body weight, it is reasonable to assume that their carbon dioxide emissions would be lower than those predicted for fattening pigs. In addition, the observed relationship between carbon dioxide emissions and body weight is consistent with previous findings (Philippe and Nicks 2015). These results suggest that the measurement chamber system used in this present study is reliable for accurately assessing carbon dioxide and methane production in pigs.

Methane is produced through the anaerobic decomposition of organic matter by archaea present in the digestive tract in pigs and is thought to be emitted via exhalation and flatus (Christensen and Thorbek 1987; Moller et al. 2004). Our continuous monitoring of gas concentration changes showed that, unlike carbon dioxide, which was emitted at relatively constant levels, methane concentrations fluctuated periodically over short periods. Fluctuations in methane concentrations may result from its rapid release followed by a decrease due to ventilation. The source of methane, whether from exhalation or flatus, remains unclear. However, it is likely to be primarily emitted as flatus, as in vitro fermentation studies on intestinal contents have shown that methane is predominantly produced in the lower gastrointestinal tract (Robinson et al. 1989).

Potential interference from feces‐based gas emissions during measurement is an important consideration. In our study, freshly excreted feces were immediately exposed to air because these conditions have been reported to minimize methane production from fecal matter (Thauer et al. 2008; Wang et al. 2021). Nonetheless, future improvements to measurement systems that can exclude or control for fecal gas emissions would enhance the accuracy of quantifying methane directly attributable to the animal.

The present study on MMPs found that the genus *Methanobrevibacter* exhibited the highest relative abundance among methane‐producing archaea in the gut. This finding is consistent with previous research on piglets and finishing pigs (Luo et al. 2017; Mi et al. 2019). In contrast, differences in the composition of methane‐producing archaea, with the exception of *Methanobrevibacter*, were observed in our study. The family Methanomethylophilaceae and genera *RumEn_M2*, *Candidatus_Methanomethylophilus*, and *Methanosphaera* were identified in the gut of MMPs other than genus *Methanobrevibacter*. Previous studies have reported *Methanosphaera* as the second‐most abundant genus following *Methanobrevibacter* in finishing pigs (Mi et al. 2019). In piglets, *Methanomassilococcus* is the second‐most abundant genus after *Methanobrevibacter* (Luo et al. 2017). Although differences in pig strains and the limitation of sample size should be considered, these findings suggest that genus *Methanobrevibacter* is the predominant methane‐producing archaea in porcine gut, although the composition of other methane‐producing genera that occupy relatively minor positions may vary based on the pig's age, breed, or other conditions. Another consideration is that although the primer set used in this study (341F–805R) has been reported to amplify major archaeal groups, it may not provide comprehensive coverage of archaeal diversity (Bahram et al. 2019). Thus, certain archaeal lineages may have been underrepresented due to primer bias. Our analysis focused on archaeal taxa that could be amplified with this primer set, and it identified *Methanobrevibacter* as the dominant group. To more fully cover archaeal diversity, especially taxa present at low abundance, future studies should consider using archaeal‐specific primers.

Methane emissions from pigs are reported to be influenced by the amount of fiber in the diet as well as the fermentation capacity of the gut (Philippe and Nicks 2015). Furthermore, the methane‐producing archaea composition in the porcine gut is modulated by the fiber content in their diet (Luo et al. 2017). Our results indicate that the relative abundance of genus *Methanobrevibacter* in the gut is positively correlated with the amount of methane produced by the pig. Based on these findings and previous studies, it is suggested that dietary fiber intake may increase the relative abundance of methanogenic archaea, potentially contributing to elevated methane emissions. However, further research is needed to more precisely assess the impact of varying levels of fiber on the composition and abundance of methane‐producing gut archaea. In addition, MMPs were used in this study due to their small size and ease of handling. However, the composition of methanogenic archaea in their gut may differ from that observed in fattening pigs, suggesting the need for future studies to investigate the relationship between methane‐producing archaea in their guts and methane emissions in fattening pigs.

One limitation of the present study is that microbial abundance was assessed solely based on the relative abundance derived from sequencing data. Although this approach allowed us to reveal a significant association between the relative abundance of the genus *Methanobrevibacter* and methane emissions, it did not account for differences in total archaeal load across individuals. Future studies should incorporate absolute quantification methods, such as quantitative PCR (qPCR) or other molecular techniques (Cisek et al. 2023; Sanchez‐Sanchez et al. 2022), to validate and refine the observed relationships between microbial communities and methane production.

In this study, a platform for monitoring GHG emissions from pigs using a photoacoustic gas monitor was developed. While the relationship between methane emissions from pigs and methane‐producing archaea in their gut has not been well understood, the findings of our study demonstrate a positive correlation between the relative abundance of genus *Methanobrevibacter* and methane emissions in MMP. These findings will help develop mitigation strategies for GHG emissions from livestock production. However, further studies are required to validate these findings in fattening pigs and to investigate the effects of feed composition on the abundance of methane‐producing archaea and methane emissions.

## Conflicts of Interest

The authors declare no conflicts of interest.
